# Infections and gender: clues for diagnosis of adrenal insufficiency—a case report and a review of the literature

**DOI:** 10.1007/s11739-024-03613-8

**Published:** 2024-06-18

**Authors:** Giacomo Grandi, Michele Di Stefano, Chiara Cebrelli, Caterina Mengoli, Antonio Di Sabatino

**Affiliations:** Department of Internal Medicine, IRCCS San Matteo Hospital Foundation, University of Pavia, P.le Camillo Golgi 2, 27100 Pavia, Italy

**Keywords:** Adrenal insufficiency, Infection, Empty sella syndrome, Gender medicine

## Abstract

The clinical presentation of adrenal insufficiency, a condition causing adrenal hormone deficiency, is characterised by non-specific symptoms and signs: consequently, an important diagnostic delay is often evident which correlates with an increased mortality. This case report shows how the clustering of some symptoms and signs may hamper the diagnostic suspicion for this condition: serum electrolyte alterations and weight loss, when associated to recurrent infections and, in female patients, an empty sella may further guide the clinician towards a diagnosis of adrenal insufficiency. Accordingly, a clinical approach taking into account gender medicine could improve the diagnostic workup.

## Background

Adrenal insufficiency is the consequence of glucocorticoid and, in some cases, mineralocorticoids deficiency. It is classified as primary, secondary, or tertiary depending on whether the impairment originates from the adrenal gland, pituitary gland, or hypothalamus respectively [1]. Typically, the aetiology correlates with the age of presentation: before the age of 20 years, the congenital forms prevail, on the contrary autoimmune conditions are entailed in patients with age between 20 and 50 years and neoplastic conditions in patients over 50 years, in relation to pharmacological, radiation or surgical treatments [1–3]. Prevalent clinical manifestations of adrenal insufficiency include weight loss, nausea, abdominal pain, myalgia, orthostatic hypotension, hyponatremia, hyperkaliemia and hypoglycaemia, the latter in particular in paediatric patients. Symptoms of associated diseases may also be present, especially in autoimmune forms. Adrenal crisis is the most severe manifestation of this condition [1–4].

Diagnosis is based on serum determination of hormone profile and additional stimulation tests to distinguish among primary, secondary, or tertiary forms. A long diagnostic delay is frequent in this condition due to the low specificity of symptoms [4, 12]. Further investigation allows the definition of the aetiology of hormone deficiency and optimises the replacement therapy with glucocorticoids and eventually mineralocorticoids, if needed.

## Objective

Recurrent infections represent a frequent complication of adrenal insufficiency and are generally considered as a consequence of steroid replacement therapy. However, infections may occur even before the recognition of the disease and, therefore, before the beginning of steroid treatment. Accordingly, they should be considered as a clinical manifestation of the disease and they could be a guide to diagnosis.

We are presenting a clinical case illustrating how recurrent infections occur in the natural history of adrenal insufficiency together with a review of the scientific literature supporting the role of adrenal insufficiency as a condition predisposing to infectious events, independently from steroid treatment.

Moreover, a high level of attention to the presence of an empty sella in female patients is mandatory. A virtuous change in the clinical and diagnostic approach to this disorder, also considering gender differences, could be useful to shorten the long diagnostic delay and reduce mortality.

## Case report

In March 2023, a 69-year-old woman from Senegal who had moved to Italy in July 2022 came to our emergency department. She complained of fever with chills, loss of appetite and vomiting during the previous week. She referred a significant weight loss, without an exact estimate, but at least 10 kg during the previous 6 months. Recently, in December 2022, she was discharged from another hospital due to pneumonia caused by *Haemophilus* spp., *Pseudomonas* spp., and *Pneumocystis jirovecii.* A diagnosis of syndrome of inappropriate anti-diuresis (SIAD), secondary to the pulmonary disease was also made and no treatment was prescribed. Moreover, a liver angioma, hepatic steatosis, a renal angiomyolipoma, asymptomatic gallstones and bilateral gonarthrosis were also present. Finally, in anamnesis, 10 full-term pregnancies were reported. The patient was exclusively taking weak opioids as analgesic therapy for her knee pain.

During the physical examination, the patient appeared severely undernourished (BMI 17.3 kg/m^2^), with dehydrated skin and oral mucosae in the absence of other objective signs. Blood tests showed a microcytic anaemia [Hb 11.4 g/dl, MCV 67.7 fl], normal blood leucocyte count with relative neutropenia and lymphocytosis, hyponatremia [132.6 mEq/l]*,* a slight increase in the CRP levels [0.74 mg/dl] with hyperferritinemia [673.6 ng/ml]. The chest X-ray showed diffuse bilateral reticulation with pulmonary consolidation in the left upper lobe. She was admitted to the infectious disease ward where lung findings were further studied with a chest CT with iodinated contrast media, which showed images of irregular bilateral consolidation with altered bronchovascular structures and some bronchiectasis. In addition, pseudo-nodular consolidations, ground-glass alterations, and areas with tree-in-bud sign were reported. On the basis of clinical, laboratory and radiological findings, sarcoidosis was ruled out. An extensive microbiological and virological exploration was carried out (Table 1), revealing positivity for *Staphylococcus aureus* and *Geotricum capitatum* in the sputum culture, as well as a latent tuberculosis infection, for which treatment with isoniazid was initiated, subsequently replaced with rifampicin due to an allergic reaction. Esophagogastroduodenoscopy (EGD) with biopsies was also performed showing no significant changes. After correction of electrolyte levels and spontaneous resolution of symptoms, the patient was discharged to home.

**Table 1:** Virological and microbiological tests

Tests	Assay	Reference values
Quantiferon test	Positive	
Mycobacterium tuberculosis DNA (induced sputum) (RT-PCR)	Negative	
Mycobacteria culture (induce sputum)	Negative	
Mycobacterium tuberculosis DNA on BAL (RT-PCR)	Negative	
Mycobacteria culture on BAL	Negative	
Culture for fungi (induced sputum)	Positive for *Geotrichum capitatum*	
Culture for Bacteria (induced sputum)	Positive for *Staphylococcus aureus*	
Culture for Bacteria on BAL	Negative	
Nasal swab for respiratory viruses:		< 45 copies/ml undetectable, < = 45 copies detectable
Adenovirus DNA (RT-PCR)	Undetectable	
Parainfluenza Virus 2 RNA (RT-PCR)	Undetectable	
Influenza virus A RNA (RT-PCR)	Undetectable	
Parainfluenza virus RNA 1/3 (RT-PCR)	Undetectable	
Influenza virus B RNA (RT-PCR)	Undetectable	
Parainfluenza virus 4 RNA (RT-PCR)	Undetectable	
Metapneumovirus RNA (RT-PCR)	Undetectable	
Coronavirus RNA OC43/HKU1 (RT-PCR)	Undetectable	
Coronavirus RNA 229E/NL63 (RT-PCR)	Undetectable	
Rhinovirus RNA (RT-PCR)	Undetectable	
Respiratory syncytial virus RNA (RT-PCR)	Undetectable	
SARS-CoV-2 rapid antigenic swab	Negative	
HIV 1–2 Antibodies	Negative	
HIV1 antigen p24	Negative	
HBsAg (ECLIA)	Negative	
HBsAb (ECLIA)	> 1000	> 10 mUI/ml presence of immunity
HBcAb (ECLIA)	Positive	
HBcAb IgM (ECLIA)	0.0	< 1 Negative
HCV Ab (ECLIA)	Negative	
*S. Pneumoniae* urinary antigen (EIA)	Negative	
*L. Pneumophila* urinary antigen (EIA)	Negative	
Urine culture	Negative	
Fecal antigen *Helicobacter pylori* (EIA)	Negative	
Stool culture Salmonella/Shigella	Negative	
Fecal antigen *Clostridium difficile* (EIA)	Negative	
Fecal parasites	Negative	
*Strongyloides* antibodies (ELISA)	0.180	< 1.1 Negative

*DNA* deoxyribonucleic acid; *RT-PCR* real-time polymerase chain reaction; *BAL* bronchoalveolar lavage; *RNA* ribonucleic acid; *HBsAg* surface antigen of the hepatitis B virus; *HBsAb* antibody against surface antigen of the hepatitis B virus; *HBcAb* antibodies against core antigen of hepatitis B virus; *IgM* immunoglobulin M; *HCVAb* antibodies against hepatitis C virus; *EIA* enzyme immunoassay; *ECLIA* electrochemiluminescence immunoassay; *ELISA* enzyme-linked immunosorbent assay

**Fig. 1:**
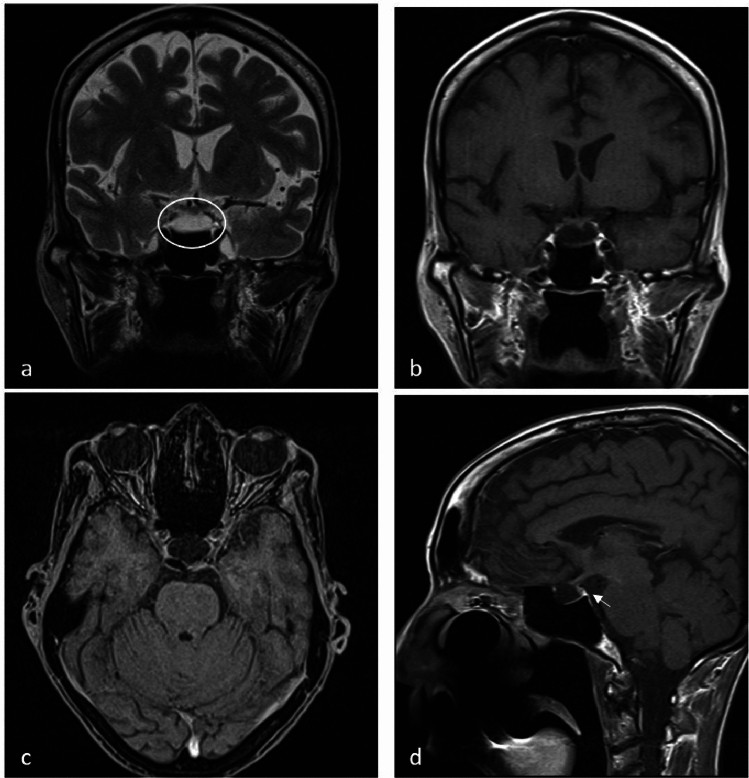
Encephalic MRI with contrast enhancement from which a picture of partial empty sella (circle) emerges with thinned and displaced parenchyma of the pituitary gland on the sella floor. A normal hyperintense neurohypophysis with the pituitary peduncle (arrow) regularly contrast enhanced, pushed back and deviated to the left. **a**: T2 sequence, coronal; **b**: T1 sequence with CE, coronal; **c**: T1 sequence with CE, axial; **d**: T1 sequence with CE, sagittal

After an asymptomatic month, she went back to the emergency department due to the occurrence of abdominal pain, vomiting, and sub-occlusive crisis. The abdomen was tense, painful with a positive Blumberg sign in the right iliac fossa. Blood tests showed hyponatremia, hypoglycaemia, normocytic anaemia, and a slight increase in the CRP levels (Table 2). Abdominal contrasted computed tomography scan showed a severe coprostasis and the absence of acute conditions. Then, she was moved to the internal medicine department. The frequent occurrence of infections and the severe weight loss made mandatory the exclusion of both an immunodeficiency status and a neoplastic condition. However, an episode of severe hypoglycaemia (14 mg/dl) with impaired cognitive status and a reduction of systemic pressure until 90/50 mmHg suggested the presence of adrenal insufficiency, confirmed by low serum cortisol and ACTH levels. Serum hormone exploration was completed and showed a panhypopituitarism (Table 2). Magnetic resonance documented a partial empty sella likely caused by the high number of pregnancies (Fig. 1). Accordingly, steroid replacement therapy was started, first with hydrocortisone, then with cortisone acetate, with a rapid symptom improvement. Electrolyte and glucose levels normalised together with the resolution of the abdominal pain. Levothyroxine was also started for the correction of hypothyroidism.

**Table 2:** Hormone and biochemical indices at diagnosis

	Blood dosage	Reference values
Cortisol	1.9 mcg/dl	4.30–22.40 mcg/dl
ACTH	9.1 pg/ml	6–57 pg/ml
Estradiol	< 11.80 pg/ml	
FSH	16.7 IU/l^a^	
LH	8.5 IU/l^a^	
fT3	1.18 pg/ml	1.18–4.20 pg/ml
fT4	5.67 pg/ml	8.00–19.00 pg/ml
TSH	1.582 mIU/l	0.40–4.00 mIU/l
GH	0.14 ng/ml	> 8 ng/ml
IGF-1	< 15 ng/ml	65.0–166.0 ng/ml
Prolactin	2.9 ng/ml	1.9–25.0 ng/ml
Sodium	124.6 mEq/l	135–153 mEq/l
Potassium	4.16 mEq/l	3.5–5.3 mEq/l
Glucose	45 mg/dl	76–100 mg/dl
Hb	10 g/dl	11.7–15.5 mg/dl
MCV	74.1 fl	82–98 fl
CRP	1.64 mg/dl	< 0.5 mg/dl
Albumin	3.3 g/dl	> 4 g/dl
Pre-albumin	5.0 mg/dl	20–40 mg/dl
Transferrin	135 mg/dl	200–360 mg/dl
Creatinine	0.51 mg/dl	0.55–1.02 mg/dl

^a^levels below normal limits because the patient is in menopause [30]

*ACTH* adrenocorticotropic hormone; *FSH* follicle-stimulating hormone; *LH* Luteinizing hormone; *fT3* free triiodothyronine; *fT4* free thyroxine; *TSH* thyroid-stimulating hormone; *GH* growth hormone; *IGF-1* insulin-like growth factor 1; *Hb* haemoglobin; *MCV* mean corpuscular volume; *CRP* c-reactive protein

Opioid-induced adrenal insufficiency was excluded by the fact that the patient assumed very low doses of this drugs and because this condition do not explain the panhypopituitarism that was discovered.

Second-level imaging allowed us to exclude the neoplastic cause of the weight loss and an immunodeficiency disorder. HIV test was negative, peripheral lymphocyte phenotype and immunoglobulin subclasses levels were normal. Primary immunodeficiency and disorders responsible for secondary immunodeficiency conditions, such as diabetes or renal failure, were excluded; the patient was not on immunosuppressive treatment. A severe malnutrition emerged from BMI, low plasma pre-albumin, albumin, transferrin, and creatinine. After the beginning of the steroid replacement therapy, the patient did not experience further infectious events in about 1 year of follow-up. She was on a stable endocrinological follow-up and attended rheumatology and orthopaedic outpatient clinics for severe gonarthrosis.

## Methods and results

To identify studies reporting the role of infectious events in adrenal insufficiency patients, we searched the electronic database PUBMED from 1960 to November 2023. Keywords used in order to broaden the spectrum of the searched condition were “adrenal insufficiency” and “infection”. A filter was applied to restrict the search to English articles and about only of human species. 1904 articles were identified. After excluding irrelevant studies and those focussed on different aspects (e.g. adrenal insufficiency caused by infections and critical illness-related corticosteroid insufficiency [CIRCI]) 35 publication were full-text review (references included) and we found 11 articles that analysed how the adrenal insufficiency increase susceptibility to infection (Table 3). Of the total articles, 8 were retrospective studies, 1 was a prospective study, 1 was a single-blind, randomised controlled trial and 1 was a cross-sectional study.

**Table 3:** The list of reviewed articles

Author	Year	Type of study	N° AI patients	Conclusion
Bergthorsdottir et al. [11]	2006	Population-based, retrospective, observational study	1675	In comparison with Swedish age-adjusted background population, the mortality from infectious diseases is five times higher than expected
Chen et al. [17]	2010	Retrospective observational study	2778	AI was rarely the sole diagnosis the patients had during the hospitalisation. Among various co-morbidities, infection, and pulmonary disease, especially pneumonia, were most common at initial AI diagnosis, during subsequent hospitalisation, and in in-hospital death
Björnsdottir et al. [16]	2013	Retrospective cohort study	1305	The patients affected by adrenal insufficiency use more antibiotics than control before and after the diagnosis
Smans et al. [15]	2013	Retrospective cohort study	390	Patients with primary adrenal insufficiency had an increased use of antimicrobial agents and hospital admissions related to infection
Isidori et al. [20]	2018	A single-blind, randomized controlled trial	89	Restoration of a more physiological circadian glucocorticoid rhythm by switching to a once-daily, modified-release regimen reduces recurrent infections
Chantzichristos et al. [7]	2017	Retrospective cohort study	226	Mortality is higher from infections and unknown cause that may be explained by untreated or inadequately treated adrenal crisis, severe hypoglycaemia or their combination
Bancos et al. [19]	2017	Cross-sectional study	42	Adrenal insufficiency is associated with significantly decreased natural killer cell cytotoxicity
Tresoldi et al. [13]	2020	Retrospective cohort study	2182	There is an increased risk of infections and antimicrobial use in PAI in the primary care setting at least partially linked to glucocorticoid treatment
Ngaosuwan et al. [6]	2021	Retrospective cohort study	6821	Mortality was increased in adrenal insufficiency. Cardiovascular disease and infections were the most frequent causes of death
Wen et al. [18]	2021	Retrospective study	11	Postoperative adrenal insufficiency is a risk factors for postoperative central nervous system infections in patients with sellar region tumours
Minnetti et al. [14]	2022	Prospective observational study	135	The disruption of endogenous GC levels, irrespective of the underlying cause, is associated with increased susceptibility to infections

*GC* glucocorticoids; *AI* adrenal insufficiency; *PAI* primary adrenal insufficiency

Fifteen Thousand Six Hundred and Fifty Four patients with adrenal insufficiency are comprehensively included in these studies published from 2006 to 2022. From this publication emerged an increased incidence of infectious events, a higher use of antimicrobial agents and also a higher mortality rate. Two possible explanations are the deficiency of cytotoxicity in natural killer cells and a non-physiological pattern in the glucocorticoids replacement therapy.

## Discussion

Adrenal insufficiency is a major public health problem. Improvements in pathophysiological knowledge, diagnostic techniques and the greater attention of clinicians have led to an increase of disease prevalence from 40 to 70 cases per million in Europe in the 1960s to 100–140 cases per million at the beginning of the current century for primary forms. The prevalence of secondary forms is 150–280 cases per million inhabitants [5]. However, there is still a significant increase in mortality, which is double in comparison with the general population, due to cardiological, oncological, infectious, and adrenal crisis-associated events [1, 6–9]. The latter events have shown an increasing incidence, estimated between 3 and 11% per year and burdened by a mortality rate of 6% [10]. A Swedish study involving 1675 patients, in comparison with Swedish age-adjusted background population, reported a mortality rate from infectious diseases five times higher than expected [11].

Despite prevalence figure and mortality, there is still an unsatisfactory diagnostic delay, especially in subacute and chronic disease: only 47% of patients receive diagnosis in the first year after the onset of symptoms and 20% after 5 years. Up to 67% of patients are evaluated by at least 3 physicians before receiving the correct diagnosis and 30% by 5 specialists. Moreover, 68% of patients initially receive a wrong diagnosis [12].

It was consistently reported that the most common clinical manifestations and laboratory findings of hypoadrenalism are asthenia (100% of reported patients), weight loss (100% of reported patients), nausea, vomiting, and abdominal pain (92% of reported patients), hyponatremia (88% of reported patients), hyperkalemia (64% of reported patients, primary adrenal insufficiency only). Serum electrolyte alterations tend to be more present during adrenal crises: this result induced the suggestion to recommend the exclusion of hypothalamus–adrenal axis impairment when unexplained hyponatremia is present, to increase diagnostic rate and reduce diagnostic delay [1, 4, 5].

Fever is considered as a possible sign, but its prevalence is not known. This could be due to alterations of pro-inflammatory cytokines regulation caused by hormone deficiency or infectious processes [11]. The latter processes were never reported as a frequent clinical manifestation of adrenal insufficiency. However, the infectious risk is significantly increased in these patients, as emerges from the case in analysis. Our patient underwent three hospitalisations for infectious processes and adrenal crisis with isolation of multiple aetiological agents over a period of about 6 months, in the absence of any other elements suggesting secondary immunodeficiency besides hypoadrenalism. Moreover, the patient showed no further infectious events after replacement therapy was initiated.

It has been shown that patients with primary adrenal insufficiency, either congenital or autoimmune, have an increased risk of developing infectious processes. Moreover, these patients have a higher rate of hospitalisation for infectious events and a higher prescription of antibiotics and antifungal drugs, before and after the diagnosis. Infections predominantly involve respiratory, urinary, and gastrointestinal tracts [13–17]. The involvement of this latter tract can be insidious as abdominal symptoms overlap adrenal crisis. Furthermore, it was shown that adrenal insufficiency is an independent risk factor for the development of central nervous system infections following neurosurgical removal of sella turcica tumours [18].

One study hypothesised the coexistence of on impaired NK T-lymphocyte function and a tendency towards decreased phagocytosis by neutrophils [19]. Part of the increased infectious risk is attributable to a high-dose steroid replacement therapy and a release pattern that do not mimicking the physiological glucocorticoid level fluctuations, altering the CLOCK gene expression pattern in immune cells [19]. The use of modified-release hydrocortisone showed the ability to reduce CD14 + /CD16- monocyte levels, increase CD14-/CD16 + monocyte levels and CD14-/CD16 + NK levels. In addition, there was a normalisation of soluble CD16 and ADAM17 levels [20].

The case of our patient, together with others [14–16, 18–20], suggests that the high frequency of infectious events may not be exclusively iatrogenic but also a direct consequence of the underlying disease. The increased use of antimicrobial agent even before the steroid therapy [16] and the presence of infection events as a co-diagnosis at the first diagnosis of the disease [17] strengthen our hypothesis. Moreover, despite the efforts to optimising steroids replacement therapy in the last years, reducing the daily dose and with a more physiological pattern of steroids exposure, infections are frequent events in these patients. Therefore, infections should be interpreted as a severe clinical manifestation of adrenal insufficiency considering the high mortality rate of infection in these patients that could be due to the increased cortisol requirements during an acute event.

However, the presence of specific alterations of the immunity, predisposing to this complication, is not completely known. Only one study reported an alteration of immune system [19, 21]. Weight loss and hyporexia, described in our patients and in most of patients with hypoadrenalism, could lead to hypothesise a role of malnutrition in the pathophysiology of secondary immunodeficiency. It was suggested to search for hypoadrenalism in the presence of electrolyte disorders, abdominal pain, weight loss, a group of symptoms highly unspecific [4, 22]. To improve the positive predictivity of clinical symptoms, clustering of some symptoms could be better. Considering hyponatremia and recurrent infections may be not sufficient to improve diagnosis, because during infectious events, besides the increase in blood glucose and plasma protein values altering sodium levels, hypovolemia secondary to ascites, renal failure, or heart failure may cause an increase of ADH secretion. In addition, pulmonary and central nervous system infections can induce SIAD. Some infections are themselves causes of primary or secondary hypoadrenalism such as *N. meningitidis, Histoplasmosis, CMV* infections and, most of all, Mycobacterium tuberculosis. The last one is a frequent cause of adrenal insufficiency, especially in developing countries, due to its possible adrenal and hypothalamic–pituitary localisation [1].

Accordingly, the association of the whole group of symptoms, including potassium and glucose alterations and gastrointestinal symptoms, with recurrent infections may substantially improve the clinical suspicion of adrenal insufficiency [23]. In the recent literature, empty sella syndrome [1, 5, 24–27] is not always counted among the numerous aetiologies of secondary hypoadrenalism. However, empty sella is a relatively frequent condition. Occasional findings during autopsy or imaging techniques vary from 5 to 35% with a higher prevalence in women than men (F:M = 5:1). Of these, approximately 20% have a partial or complete alteration of pituitary hormone secretion. While forms of secondary empty sella should be easily suspected with a careful anamnestic investigation, primary empty sella may be unrecognised. The main risk factors are endocranial hypertension, systemic hypertension, obesity, and changes in pituitary size during physiological conditions such as pregnancies, lactation, and menopause [28, 29].

These latter conditions are strictly associated with the female gender, possibly explaining the different incidence between males and females. Thus, especially in female, empty sella syndrome should be counted in the possible causes of secondary adrenal insufficiency. Obesity is a condition with a huge worldwide prevalence and its possible complications cannot be underestimated. Multiple pregnancies, on the other hand, are nowadays more frequent in developing countries, but due to significant migration flows, they should be considered also in highly industrialised countries. This point emerges from the clinical case in analysis where, thanks to a careful anamnesis, it was possible to rule out other causes of empty sella, and this significant finding guided the diagnostic process.

## Conclusions

Considering the increasing prevalence, diagnostic delay, and persistently high mortality, there is a need to improve the clinical approach to hypoadrenalism. This can be done by enhancing the pathophysiological knowledge, in particular clarifying whether alterations of the immune system are associated with this condition. Focussing on infectious processes as elements of the natural history of this condition the clinicians’ approach should change, leading to suspect adrenal insufficiency in patients with recurrent infectious events especially when associated with signs or symptoms such as hyponatremia, hyperkaliemia, hyporexia, and weight loss. The use of drugs that mimic daily fluctuations of glucocorticoid hormones could reduce the infectious risk and the associated excess in mortality. In addition, patient education should be strengthened to improve personal hygiene and reduce the risk of urinary tract infections. Strategies aimed at the reduction of the risk of respiratory system infections should be also suggested, e.g. the use of face masks in enclosed places together with *Streptococcus pneumoniae, Haemophilus influenzae, influenza virus and SARS-CoV-2* vaccinations.

Lastly, empty sella is a highly prevalent condition with risk factors hugely present in western population, particularly in the female sex. To recognise the association between empty sella and hypoadrenalism could be another way to improve diagnose and shorten diagnostic delay.
